# A scoping review of force plates in female soccer: The utility, existing practice and identification of knowledge Gaps

**DOI:** 10.1371/journal.pone.0351121

**Published:** 2026-06-15

**Authors:** Jack T. Fahey, Francisco Javier Robles-Palazón, Paul Comfort, Nichoals Ripley

**Affiliations:** 1 Directorate of Psychology and Sport, School of Health and Society, University of Salford, Salford, United Kingdom of Great Britain and Northern Ireland; 2 Department of Football Medicine and Science, Manchester United Football Club, Carrington Training Complex, Manchester, United Kingdom; 3 Department of Physical Activity and Sport, Faculty of Sport Sciences, Campus of Excellence Mare Nostrum, University of Murcia, Murcia, Spain; 4 School of Medical and Health Sciences, Edith Cowan University, Joondalup, Western Australia; Portugal Football School, Portuguese Football Federation, PORTUGAL

## Abstract

Female soccer has grown exponentially with increasing funding, participation and competition. Force plates enable high resolution force-time assessment using isometric and dynamic tasks for acute or longitudinal monitoring, and profiling performance and injury risk. However, more research has been conducted in male soccer players compared to females with the utility of force plates in female soccer yet to be reviewed. The purpose of this scoping review is to review existing research using force plates in female soccer describing the utility of force plates, methods used, reported metrics and to identify gaps in our current understanding. A literature search was conducted using PubMed, Google Scholar, Medline, and Cinahl using the following search terms: “girl*” OR “female*” OR “women*” AND “football*” OR “soccer*” AND “force plat*” AND “ jump” OR “isometric” AND (“multi joint*” OR “single joint*” OR “plantar flex*” OR “knee*” OR “hamstring*” OR “knee flex*” OR “hip exten*” OR “posterior chain*” OR “drop*” OR “rebound*” OR “stiffness*” “power*” OR “strength*” OR “counter*movement*” OR “dominant*” OR “non dominant*”). Sixty-three studies were eligible with most studies investigating senior elite players during dynamic tasks. The bilateral countermovement jump was the most common test followed by pre-planned change of direction and the isometric mid-thigh pull. Force plates were mostly used for performance assessment/ sex comparison research designs, followed by acute monitoring. Peak force was the most reported metric across all studies and jump height was most reported in studies investigating jumping. Most researchers used force plates in laboratory settings relating to change of direction and landing tasks with limited research conducted in applied settings and at youth level. Therefore, future researchers should focus on field-based assessments aiming to provide benchmarks and prospectively track players for injury as to support the health and performance of female soccer players at all levels of competition.

## 1. Background

Since the first female soccer world cup in 1981 [1], female soccer has grown exponentially due to the success of global, continental and national governing bodies specific women’s strategies to develop opportunities, provisions and performance from grassroots to elite and international levels [1,2–4,5–6]. Since 2019, global participation has increased by approximately 24% [6], with increased provisions and professionalisation of elite playing standards [2]. This has consequently led to an increase in the physical match demands which increase with age [7] and competition level [8]. Research in female soccer has also increased but still lacks behind the volume conducted in male soccer which mainly focuses on elite senior players, in particular injury incidence and strength and conditioning practises [9,10,11]. Unfortunately, injury risk is greater in female athletes compared to male athletes, particularly for non-bone-related injuries (i.e., ligament or soft tissue injuries) such as anterior cruciate ligament injury (ACL) [12,13,11,14,15,16]. In females, injury incidence is greater in matches (19.2 per 1000 hours of exposure) compared to training (3.5 per 1000 hours of exposure) [12,13], with the most common injury location being the knee [9]. Robles-Palazón et al. [14] also reported greater injury incidence in youth female soccer compared to youth male counterparts (6.77 vs 5.70 per 1000 hours of exposure, respectively). This may be due to less sport science and medical provisions in female soccer than those in male soccer despite the rapid growth [17], and recommendations are made for more research supporting female soccer players health and performance [18,19].

Advancements in sports technology (i.e., force plates) enable high resolution assessment of ground reaction forces. Force plates include piezoelectric sensors or strain gauges which measure the force applied to the platform, also referred to as ‘*ground reaction force’*, and relate to Newtons third law (i.e., every action has an equal and opposite reaction) [20]. Laboratory grade force plates can collect tri-planar force data (i.e., in the vertical, anterior-posterior and medio-lateral components), while more commercially available and portable force plates are typically uniaxial, only collecting the vertical component. Force plates can be used to determine performance in relatively simplistic multi-joint dynamic tasks such as jump performance, enabling the calculation of several variables including jump outcome (i.e., jump height), jump drivers or kinetics (i.e., peak and average forces) and jump strategy (i.e., temporal aspects) [21,22]. Isometric tasks can also be performed with force plates across multi-joint (i.e., mid-thigh pull (IMTP)) or single joint assessments (i.e., 90–90 hamstring test [supine with 90º of hip and knee flexion), enabling the determination of maximal and rapid isometric force production [23,24,25]. Force plates can also be combined with other technology such as three-dimension (3D) motion capture to observe more dynamic tasks such as landing [26,27,28,29,30] and changes of direction [31–33], enabling the determination of joint loads (i.e., knee joint) through inverse dynamics and which when combined with identification of body segment position can elucidate factors related to injury risk [33–35,29,30].

Commercially available and portable force plates have increased in popularity with approximately 50% of strength and conditioning coaches in men’s soccer using force plates to monitor neuromuscular function [36]. A common application of force plates is to monitor changes in performance as a proxy of neuromuscular fatigue, with the bilateral countermovement jump (CMJ) being the most commonly applied test [37,38], likely due to the time-efficiency and minimal resource needed to conduct the test. Guthrie et al. [38] reported jump height as the most frequently reported metric along with peak power, relative peak power, flight time, flight time to contraction time ratio and reactive strength index [38]. However not all studies included in this review used force plates or calculated jump height using the same calculation (i.e., flight time or impulse-momentum theorem). Also, peak power during CMJ assessments (or other jump variants such as unilateral CMJ) using force plates does not reflect physiological power generated by the athletes’ muscles nor jumping performance, despite strong correlations between peak power and jump height [21]. Rather, power is a compound variable calculated as the product of instantaneous ground reaction force and instantaneous velocity, meaning that the correlation is artificially inflated by the near-perfect correlation between jump height and velocity at take-off [21]. Badby et al. [37] reviewed 30 studies which used force plates to monitor neuromuscular fatigue across a mixture of male and female sports including team sports (i.e., handball, rugby codes, soccer), individual sports (i.e., runners and triathletes), university sports students and tactical populations. A similar observation was made with the bilateral CMJ being the most common test, with ninety metrics reported in the review and the top five being: jump height, concentric phase time, eccentric phase time, peak force and peak power [37]. The current authors wish to acknowledge that concentric and eccentric used in the studies reviewed by Badby et al. [37] refer to the propulsion braking phases respectively. Despite the simplicity of jump height (i.e., outcome), force plates provide further metrics relating to the person (i.e., body mass), driver (i.e., braking and propulsive force) and strategy (i.e., time to take-off) which offer greater insights for practitioners when acute or chronic monitoring of performance [39,40,22]. As jump height can be maintained due to a reduction in body mass or an alteration in jump strategy (i.e., increased time to take-off) to achieve the appropriate relative net propulsive impulse [40], highlighting the benefits of tracking more than jump height alone. Badby et al. [37] also reported inconsistencies in reporting the data analysis procedures, for example calculation of CMJ metrics and inaccurate terminology (i.e., concentric and eccentric). Recommendations for interpreting the CMJ force time curve have been recommended by McMahon et al. [41], as well as terminology relating the biomechanical principles (i.e., braking and propulsive) rather than muscle actions (i.e., concentric and eccentric) [42]. Other researchers have used multi-joint (i.e., mid-thigh pull) [43] and single joint [44,45,46] isometric tests to acutely or longitudinally monitor neuromuscular function and fatigue. These tests cause less fatigue and muscle soreness than supramaximal and dynamic tasks (i.e., Nordic Hamstring Exercise) and allow regular monitoring of maximal and rapid isometric force production capability [47]. Another application of force plates is to benchmark athletes’ physical performance beyond outcome metrics. McMahon et al. [48] reported CMJ normative data for rugby league forwards and backs, using T-scores [(z-score x 10 (+ 49))] and a traffic light system with performance bands and descriptors for ease of interpretation for end users [50]. This information can help practitioners design physical development programs, guide return to play and support talent identification.

A well-established risk factor for future injury is previous injury [51,11], however this is non-modifiable and there may be instances when athletes are without previous injury (i.e., youth athletes) [14]. Other risk factors include, but are not limited to maturation status (especially during peak height velocity) [52,14], body mass index [53], joint hypermobility [54], reduced lower limb relative strength [55,56], movement characteristics [57], dynamic knee valgus during landing tasks [58,59], excessive training and/or match exposure [55,54], nutritional considerations [60] and psychological factors [61]. Despite the growing research in female soccer, which lags in comparison to the volume of male soccer research [10,19], practitioners and researchers would benefit from designing and assessing interventions that support female soccer players health and performance [18,19], and reduce injury risk [56]. Di Paolo et al [57] combined force plates with two and 3D motion capture and reported greater kinematic differences during a pre-planned 90º cutting task for female soccer players who sustained an ACL injured over a two-season prospective study. Jones et al. [33,34] reported modifiable technical factors and determinants for change of direction in female soccer players. Although these studies provide valuable insights for practitioners, who can design programs to address modifiable risk factors such as technique modification [62] and relative lower limb strength [63], these studies are laboratory based which may not always be available for applied practitioners due to cost, equipment availability and expertise for data collection and interpretation. Force plates have been used in applied settings during return to play following ACL injury [64] and hamstring injuries [65–67]. Baumgart et al. [64] reported greater kinetics during in the jumping and landing in bilateral and unilateral jumps in the operated limb following an ACL injury, whereas Taberner et al. [65–67] used force plates to monitor isometric posterior chain peak force and asymmetry following a hamstring injury and surgery. Although peak force is a reliable metric to monitor during isometric hamstring assessments [25,46,68], rapid force production is also an important quality that practitioners could assess given the sprinting-based injury occurrence [69], and importance of the hamstring musculature to produce high forces rapidly during the terminal swing phase of sprinting [70,71].

Considerations and recommendations pertaining to force plate set up and standard operating procedures have established across dynamic [72–74] and isometric tasks [72,75–80]. Bishop et al. [39,81] provided recommendations for selecting metrics for the CMJ and drop jump test when performance profiling, fatigue monitoring and return to play. One of the metrics recommended for during return to play was landing impulse [39], however this equates to propulsive impulse and can be determined from jump (or fall) height. Fahey et al. [82] reported poor reliability of landing metrics, which could also be influenced by cueing [83]. Practitioners are encouraged to consider the constituent parts to impulse and ratio metrics and to ensure appropriate test-retest reliability of the metrics selected in order to determine a suitable minimal detectable change. Test-retest reliability for the bilateral and unilateral CMJ, and countermovement rebound jump (CMJ-R) in female youth soccer players [82], and appears stable following six-weeks of combined strength and jump training [84], however reliability has been suggested to improve with maturation [85]. The findings of a recent systematic review and meta-analysis identified benchmarks for physical performance testing in female soccer across a range of physical qualities (e.g., aerobic fitness, linear sprinting speed and CMJ), however multiple limitations identified by the authors included a lack of consistency across testing protocols, insufficient sample sizes and misrepresentation of different performance levels [86]. It is essential that consistent testing procedures are maintained to avoid outcome measures (e.g., jump height) being influenced by different strategies [87,21,88]. As female soccer continues to grow with more sport science and medical provisions, coaching, applied research and advancements in technologies such as portable force plates, the physical match demands appear to increase with age [89,7] and competition [8]. However, this means that practitioners will be under scrutiny to identify modifiable injury risk factors [31,33–35], and design interventions to support female health and performance of female soccer players especially given the greater injury risk in female soccer players compared to males [18,19]. To date, the research in female soccer using force plates has yet to be synthesised, which would outline current knowledge and existing gaps. Therefore, this scoping review was designed to synthesise the existing research using force plates in female soccer players and to identify existing knowledge and gaps to guide future research.

## 2. Methods

### 2.1. Study design

A 27-item checklist defined within the Preferred Reporting Items for Systematic Reviews and Meta-Analyses extension for Scoping Reviews (PRISMA-ScR) was used in this scoping review (S1 File). This was selected as a basis for reporting systematic reviews of randomised trials [90]. A review protocol was not pre-registered for this review due to exploratory nature of this scoping review.

### 2.2. Literature Search

A systematic, computerised literature search using PubMed, Google Scholar, Medline, and CINAHL was conducted. Search terms with controlled vocabulary and key words were combined using Boolean logic (AND [between categories], OR [within categories]), relating to isometric assessment, force plates, and hamstring function or strength. Search terms were split across multiple search input boxes in PubMed, Medline, and CINAHL (S2 File), whereas all search terms were listed in the single search box in Google Scholar. The search terms are listed below:

(((“girl*” OR “female*” OR “women*” AND “football*” OR “soccer*”) AND (“force plat*”)) AND (“jump” OR “isometric”)) AND (“multi joint*” OR “single joint*” OR “plantar flex*” OR “knee*” OR “hamstring*” OR “knee flex*” OR “hip exten*” OR “posterior chain*” OR “drop*” OR “rebound*” OR “stiffness*” “power*” OR “strength*” OR “counter*movement*” OR “dominant*” OR “non dominant*”))

The results from each database were imported into Covidence for abstract, keywords and full text screening and was identical across all databases including Google Scholar, despite the volume of non-academic research that can be included. Any non-academic research outputs were excluded manually during the screening process by JF and FJRP. No restrictions were placed on age but all participants must be representative of tiers 2–5 according to McKay et al. [91], which due to the criteria (i.e., female soccer players) that participants must be participating in soccer, tiers 0–1 could not be included. In the case where researchers did not categorise performance level, the participant inclusion criteria was cross-referenced according to McKay et al. [91]. The search was conducted on 24^th^ February 2024 and updated on 11^th^ July 2025 and 31^st^ October 2025 to check for new references. Reference lists were also examined for further potentially relevant studies.

### 2.3. Inclusion and Exclusion Criteria

A comprehensive inclusion and exclusion criteria were determined by the authors to ensure the initial search was successful. As proposed by the Joanna Briggs Institute framework [92], these criteria followed the elements of population, concept, and context (Table 1). Investigations using force plates and single joint isometric assessment were eligible for full text review.

**Table 1:** Inclusion and exclusion criteria based on the Joanna Briggs Institute framework [92].

Category	Include	Exclude
Participant	Female/ women soccer/soccer players Age range = 8–50 years Soccer players considered between tiers 2–5 as defined by McKay et al. [91] Physically abled and disabled players	Multi-sport athletes or a combined athlete pool which does not separate female soccer players Retired soccer players
Concept	Force assessment conducted by single or multiple force plates Single or multi-joint assessments Isometric or dynamics tests	Force assessment measured without force plates, including contact matts, electronic transmitting systems (i.e., Opto Jump), hand-held dynamometer, isokinetic dynamometer only
Context	Study aim: reliability, acute monitoring, longitudinal monitoring, descriptive or normative data, performance assessment or sex comparison, prospective or retrospective injury risk	Other study and assessment purposes
Sources	Study design: experimental, cross-sectional, observational English language	Study Design: non-experimental designs (reviews) Non-peer reviewed publication including thesis and conference abstract Non-English language Full text not available

### 2.4. Quality Assessment

The methodological quality of articles was assessed (JF & FJRP) using a mixed methods appraisal tool (MMAT) [93] with any conflicts resolved by NR, and the findings provided for descriptive purposes only. This tool allows critical appraisal mixed method study designs including qualitative research, randomized controlled trials, non-randomized studies, quantitative descriptive studies, and mixed methods studies, allowing any methodological inconsistencies to be identified. For quantitative descriptive studies, risk of non-response bias was considered low (i.e., yes) when a minimum of ≥80% of all trials were completed.

## 3. Results

### 3.1. Results Search

**Fig 1:**
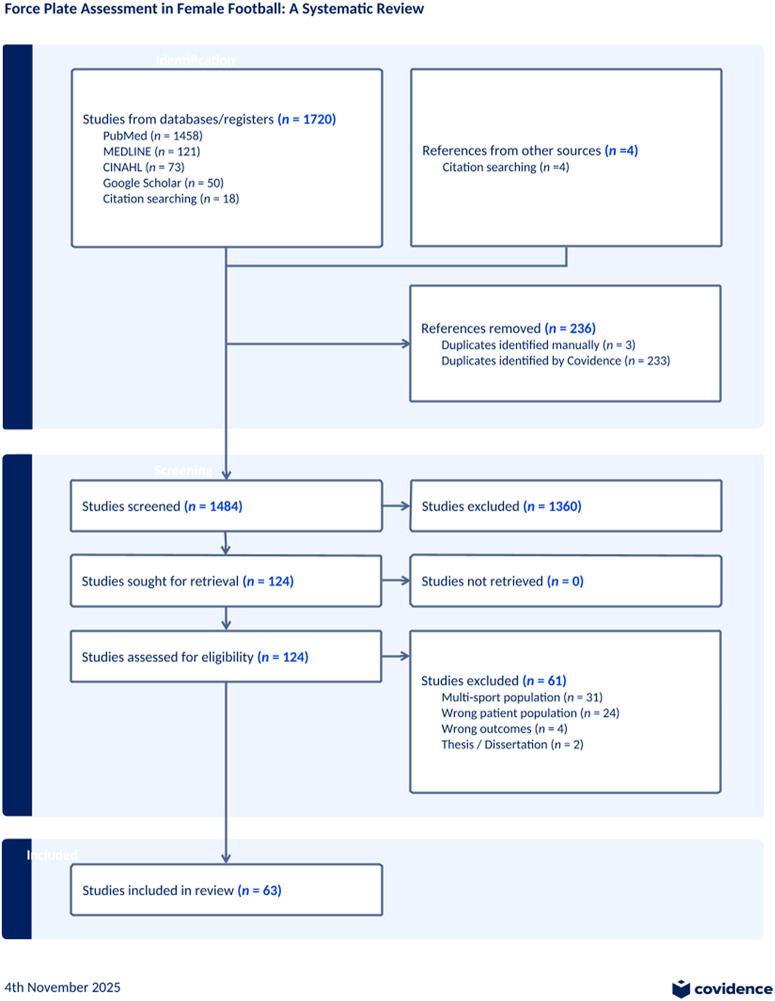
Preferred Reporting Items for Systematic reviews and Meta-Analyses extension for Scoping Reviews (PRISMA-ScR) flow diagram of included studies.

Following searches (24^th^ February 2024, 11^th^ July 2025 and 31^st^ October 2025), a total of 1720 studies were identified through database and reference searches. After title, abstract and full text screening 1360 studies were excluded, leaving sixty-three studies eligible for data extraction (Fig 1).

### 3.2. Study Demographics

**Fig 2:**
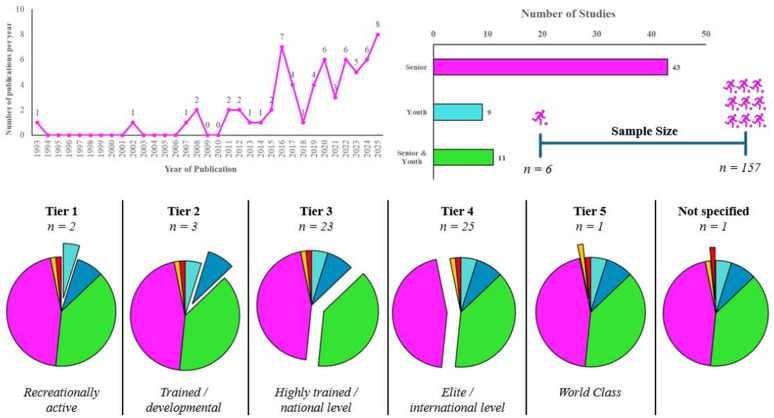
An overview of the number of publications and study demographics.

The number of publications has steadily increased since the first study that included force plate assessments in female soccer players in 1993, with fifty of the sixty-three studies included in this review being published in the last decade. Forty-three studies included senior female soccer players (i.e., > 18 years of age), nine studies included youth (i.e., < 18 years of age) female soccer players, and eleven studies included a combination of both youth and senior female soccer players. The number of female soccer players per study ranged from 6 [94,95] to 157 [49,96,97] with players performance calibre (determined by McKay et al. [91], ranging from trained/developmental (tier 2) to world class athletes (tier 5) with one study unable to specify [94]. One study categorised youth players by maturity status [96], two studies provided maturity status but categorised players by chronological age [49,43] along with all other studies of youth female soccer players. A summary of the participant characteristics is displayed in Fig 2 and Table 2.

**Table 2:** Summary of study design and demographics.

Reference	Study Design	Population & Sport	Performance Caliber	Sample Size	Familiarisation	Warm Up	Age (years)	Body Mass (kg)	Height (m)
Abbott et al. [98]	Cross-sectional observational	Professional soccer players	Tier 4	16	Yes	Yes	26.0 ± 4.0 years	67.9 ± 6.4 kg	1.70 ± 0.1 m
Andersson et al. [99]	Cross-sectional observational	International senior soccer players	Tier 4	22	Yes	Yes	Active group – 22.6 ± 4.2 years Passive group – 21.6 ± 2.6 years	Active group – 63.3 ± 7.1 kg Passive group – 65.0 ± 4.6 kg	Active group – 1.67 ± 0.6 m Passive group – 1.67 ± 0.5 m
Barber et al. [23]	Cross-sectional observational	Elite youth and senior soccer players	Tier 4	20	NS	Yes	20.7 ± 4.7 years	62.8 ± 7.0 kg	1.68 ± 0.6 m
Barnfield et al. [100]	Cross-sectional observational	Elite male and soccer players	Tier 4	8	NS	Yes	19-22 years (range)	Female = 60.1 kg. Male = 87.3 kg	Female = 1.64 m. Male = 1.84 m
Bishop et al. [101]	Cross-sectional observational	Elite youth soccer players	Tier 4	18	Yes	Yes	15.9 ± 0.8 years	57.8 ± 7.0 kg	1.65 ± 0.7 m
Busko et al. [102]	Cross-sectional observational	Deaf soccer players	Tier 2	13	NS	NS	Deaf trained −23.6 ± 4.3 years	Deaf trained −62.6 ± 8.4 kg	Deaf trained – 1.64 ± 0.5 m
Butler et al. [103]	Prospective observational	Recreationally active soccer players	Tier 2	28 (14 female)	NS	NS	Between 18–30 years	NS	NS
Cabarkapa et al. [104]	Prospective observational	Semi-professional soccer players	Tier 3	71 (26 female) 10 – injured 16- non-injured	NS	Yes	Injured group = 22.8 ± 5.9 years Non-injured groups = 24.5 ± 3.9 years	Injured group = 67.4 ± 9.1 kg Non-injured groups = 64.0 ± 8.7 kg	Injured group = 1.72 ± 0.7 m Non-injured groups = 1.70 ± 0.7 m
Condello et al. [105]	Cross sectional-observational	NCAA III soccer players	Tier 3	26 (12 female)	NS	Yes	21.0 ± 2.7 years	61.9 ± 6.2 kg	1.67 ± 0.4 m
Cortes et al. [106]	Cross-sectional observational	NCAA I soccer players	Tier 4	13	NS	Yes	19.3 ± 0.9 years	61.3 ± 5.6 kg	1.68 ± 0.1 m
Cortes et al. [107]	Cross-sectional observational	NCAA I soccer players	Tier 4	18	NS	Yes	19.0 ± 0.9 years	62 ± 5 kg	1.66 ± 0.1 m
Cuthbert et al. [108]	Repeated measures cross-sectional	Women’s Super League female soccer players	Tier 4	29	Yes	Yes	20.7 ± 4.7 years	64.4 ± 6.7 kg	1.69 ± 0.6 m
Della Villa et al. [109]	Cross-sectional observational	Recreational and elite soccer players	Tiers 2 and 4	34 (16 female)	NS	Yes	22.8 ± 4.1 years (pooled)	68.6 ± 12.7 kg (pooled)	1.75 ± 0.1 m (pooled)
Di Paolo et al. [110]	Cross-sectional observational	Recreational and elite soccer players	Tiers 2 and 4	34 (16 female)	NS	Yes	22.8 ± 4.1 years (pooled)	68.6 ± 12.7 kg (pooled)	1.75 ± 0.1 m (pooled)
Di Paolo et al. [57]	Prospective observational	Soccer players	Tier 3	16	NS	Yes	21.4 ± 4.3 years	58.1 ± 4.4 kg	1.67 ± 0.7 m
Emmonds et al. [49]	Cross-sectional observational	Youth soccer players	Tier 4	147	Yes	Yes	Maturity offset U10 = −2.5 ± 0.5 years U12 = −0.9 ± 0.7 years U14 = 1.0 ± 0.7 years U16 = 2.3 ± 0.5 years	U10 = 29.7 ± 5.1 kg U12 = 37.7 ± 8.0 kg U14 = 50.1 ± 7.6 kg U16 = 56.8 ± 7.2 kg	U10 = 1.35 ± 0.8 m U12 = 1.47 ± 0.9 m U14 = 1.59 ± 0.7 m U16 = 1.64 ± 0.6 m
Emmonds et al. [97]	Cross-sectional observational	Youth soccer players	Tier 4	157	NS	Yes	U10 = 9.3 ± 0.6 years U12 = 11.4 ± 1.0 years U14 = 13.2 ± 0.7 years U16 = 15.1 ± 0.6 years	U10 = 29.7 ± 5.1 kg U12 = 37.6 ± 8.0 kg U14 = 50.1 ± 7.6 kg U16 = 56.8 ± 7.2 kg	U10 = 1.35 ± 0.8 m U12 = 1.47 ± 0.9 m U14 = 1.59 ± 0.7 m U16 = 1.64 ± 0.6 m
Emmonds et al. [111]	Cross-sectional observational	Women’s Super League female soccer players	Tier 4	10	Familiarisation trials permitted	Yes	25.4 ± 7.0 years	62.6 ± 5.1 kg	1.67 ± 0.5 m
Emmonds et al. [96]	Cross-sectional observational	Youth soccer players	Tier 4	157	NS	Yes	Maturity offset (years from PHV) −2.5 = 9.2 ± 0.6 years −1.5 = 10.7 ± 0.6 years −0.5 = 11.9 ± 0.3 years +0.5 = 12.8 ± 0.7 years +1.5 = 14.0 ± 0.7 years +2.5 = 15.2 ± 0.7 years	Maturity offset (years from PHV) −2.5 = 28.3 ± 4.5 kg −1.5 = 33.4 ± 3.8 kg −0.5 = 40.5 ± 4.9 kg +0.5 = 49.0 ± 5.0 kg +1.5 = 54.9 ± 5.1 kg + 2.5 = 57.5 ± 7.5 kg	Maturity offset: −2.5 = 1.32 ± 0.6 m −1.5 = 1.42 ± 0.4 m −0.5 = 1.51 ± 0.5 m +0.5 = 1.57 ± 0.5 m +1.5 = 1.62 ± 0.4 m +2.5 = 1.66 ± 0.7 m
Emmonds et al. [43]	Cross-sectional observational	Youth soccer players	Tier 4	113	NS	Yes	U10 = 9.3 ± 0.5 years U12 = 11.3 ± 0.5 years U14 = 13. ± 0.7 years U16 = 15.1 ± 0.7 years	U10 = 29.6 ± 4.8 kg U12 = 38.2 ± 8.2 kg U14 = 49.3 ± 7.3 kg U16 = 56.9 ± 6.7 kg	U10 = 1.35 ± 0.7 m U12 = 1.48 ± 0.9 m U14 = 1.59 ± 0.8 m U16 = 1.65 ± 0.6 m
Fahey et al. [82]	Cross-sectional observational	Youth and senior soccer players	Tiers 3–4	27	Yes	Yes	15.4 ± 1.6 years	59.2 ± 2.4 kg	1.66 ± 0.5 m
Fahey et al. [84]	Observational study	Youth soccer players	Tiers 3–4	28	Yes	Yes	13.7 ± 1.1 years	53.3 ± 8.8 kg	1.62 ± 0.5 m
Fahey et al. [112]	Observational study	Youth soccer players	Tiers 3–4	32	Yes	Submaximal trials	13.9 ± 1.1 years	53.6 ± 8.2 kg	1.63 ± 0.5 m
Fahey et al. [113] Muscles	Cross-sectional observational	Youth and senior soccer players	Tiers 3–4	96 (19 = stronger groups vs 23 = weaker group)	Yes	Yes	14.1 ± 2.3 years	55.0 ± 10.3 kg Stronger = 56.5 ± 8.7 kg Weaker = 52.2 ± 8.6 kg	1.61 ± 1.0 m
Ferreria et al. [114]	Randomized, parallel, and double-blind	Soccer players	Tier 3	37 (7 female)	NS	NS	23.8 ± 1.9 (pooled)	68.2 ± 1.1 (pooled)	1.74 ± 0.6 m (pooled)
Fu et al. [115]	Observational study	Elite soccer players	Tier 4	19	Yes	Yes	20.7 ± 1.1 years	57.8 ± 7.2 kg	1.65 ± 0.6 m
Gauffin et al. [94]	Cross-sectional observational	Soccer players	NS	6	NS	NS	22.0 ± 4.0 years	59.0 ± 7.0 kg	NS
Goulart et al. [116]	Cross-sectional observational	Professional Brazilian soccer players	Tier 3	10	Yes	Yes	25.1 ± 5.9 years	58.9 ± 6.2 kg	1.62 ± 0.6 m
Greska et al. [117]	Non-randomized observational	NCAA I soccer players	Tier 4	12	Yes	NS	19.2 ± 0.8 years	60.2 ± 6.5 kg	1.67 ± 0.1 m
Harato et al. [118]	Cross-sectional observational	Collegiate soccer players	Tier 3	42 (8 soccer players)	NS	Warm up trials	18.1 ± 0.1 years	54.4 ± 2.6 kg	1.60 ± 0.05 m
Haugen et al. [119]	Longitudinal observational	Youth and senior soccer players	Tiers 3–4	194	Familiarisation trials permitted	Yes	22.0 ± 4.1 years	63.0 ± 5.6 kg	NS
Haugen et al. [120]	Cross-sectional observational	National team soccer players	Tier 4	95	NS	Yes	24.0 ± 4 years	65.0 ± 6.0 kg	NS
Ishida et al. [121]	Cross-sectional observational	NCAA I players	Tier 4	12	Yes	Yes	20.7 ± 2.3 years Range 19–22 years	64.4 ± 7.2 kg	1.65 ± 0.6 m
Ishida et al. [122]	Cross-sectional observational	NCAA Division I soccer players	Tier 4	14	Yes	Yes	20.7 ± 1.3 years	63.3 ± 7.0 kg	1.65 ± 0.6 m
Jones et al. [31]	Cross-sectional observational	Soccer players	Tier 3	19	Yes	Yes	21.7 ± 4.3 years	60.5 ± 6.1 kg	1.67 ± 0.07 m
Jones et al. [33]	Cross-sectional observational	Elite and sub elite soccer players	Tiers 2–3	26	Yes	NS	21.0 ± 3.2 years	59.1 ± 6.8 kg	1.68 ± 0.07 m
Jones et al. [34]	Cross-sectional observational	Soccer players	Tier 3	27	Yes	NS	21.0 ± 3.8 years	60.0 ± 7.2 kg	1.67 ± 0.07 m
Jones et al. [32]	Cross-sectional observational	Soccer players	Tier 3	22	Yes	NS	21.0 ± 3.1 years	58.9 ± 7.3 kg	1.68 ± 0.07 m
Jones et al. [35]	Cross-sectional observational	Soccer players	Tier 3	18	Yes	Yes	21.6 ± 4.3 years	60.3 ± 6.3 kg	1.67 ± 0.07 m
Khalid et al. [95]	Cross-sectional observational	University soccer players	Tier 2	6	Yes	Yes	19.3 ± 2.0 years	55.4 ± 9.6 kg	1.61 ± 0.4 m
Lesinski et al. [123]	Cross-sectional observational	Sub-elite youth soccer players	Tier 2	19	One familiarisation trial permitted	Yes	14.7 ± 0.6 years	57.6 ± 6.2 kg	1.66 ± 0.5 m
Loturco et al. (106g)	Cross-sectional observational	Elite soccer players	Tier 4	16	Yes	Yes	23.0 ± 3.8 years	60.2 ± 7.3 kg	1.65 ± 0.6 m
Louder et al. [124]	Cross-sectional observational	NCAA I soccer players	Tier 4	44 (12 female soccer players)	NS	NS	19.9 ± 1.1 years	62.2 ± 5.7 kg	1.61 ± 0.8 m
Malloy et al. [125]	Cross-sectional observational	NCAA I soccer players	Tier 4	23	NS	NS	19.4 ± 0.8 years	61.0 ± 4.0 kg	1.68 ± 0.5 m
Orloff et al. [126]	Cross-sectional Observational	NCAA III soccer players	Tier 3	23 (12 female)	Familiarisation trials permitted	Yes	20.2 ± 1.2 years	NS	NS
Rath et al. [127]	Cross-sectional Observational	NCAA II soccer players	Tier 3	12	One familiarisation trial permitted	NS	19.4 ± 1.4 years	64.1 ± 4.8 kg	1.64 ± 0.05 m
Ripley et al. [46]	Repeated measures cross-sectional	Women’s Super League female soccer players	Tier 4	23	Yes	Submaximal trials	20.7 ± 4.7 years	64.4 ± 6.7 kg	1.69 ± 0.6 m
Ripley et al. [68]	Cross-sectional Observational	Elite footballers	Tier 4	33	Familiarisation trials permitted	Yes	18.7 ± 3.7 years	62.8 ± 5.5 kg	158.3 ± 5.9 m
Ripley et al. [25]	Cross-sectional Observational	Elite soccer players	Tier 4	21	NS	Yes	20.7 ± 4.7 years	62.8 ± 7.0 kg	1.68 ± 0.6 m
Romero-Moraleda et al. [128]	Cross-sectional longitudinal observational	Elite soccer players	Tier 5	20	Yes	Yes	24.9 ± 3.45 years	62.2 ± 6.4 kg	1.68 ± 0.6 m
Romero-Moraleda et al. [129]	Cross-sectional longitudinal observational	Elite soccer players	Tier 4	15	1RM and RPE NS for CMJ	Yes	24.0 ± 3.0 years	59.0 ± 9.2 kg	1.68 ± 0.11 m
Seraphin et al. [130]	Cross-sectional Observational	National Women Soccer League Soccer players	Tier 4	28	NS	Yes	27.8 ± 5.1 years	66.2 ± 6.7 kg	NS
Siegel et al. [131]	Cross-sectional Observational	German soccer players	Tier 3	21	Low intensity trial	Yes	20.5 ± 4.4 years	65.2 ± 7.5	1.69 ± 0.05 m
Sharma & Singh [132]	Cross-sectional Observational	University level soccer players	Tier 3	96 (24 footballers) Sexes NS	NS	Yes	21.6 ± 1.2 years	52.9 ± 3.5 kg	1.60 ± 0.40 m
Smith et al. [133]	Cross-sectional Observational	NCAA I and NCAA III soccer players	Tiers 3–4	34 (19 NCAA I, 15 NCAA III)	NS	NS	Not specified	NCAA I = 63.3 ± 7.4 kg NCAA III = 62.9 ± 10.5 kg	NCAA I = 1.67 ± 0.5 m NCAA III = 1.63 ± 0.6 m
Steig et al. [134]	Cross-sectional Observational	Collegiate soccer players	Tier 3	17	NS	Yes	18.9 ± .7 years	66.1 ± 6.4 kg	169.4 ± 5.3 kg
Struzik & Pietraszewski [135]	Cross-sectional Observational	Polish national soccer players	Tier 4	14	Yes	Yes	20.6 ± 4.1 years	59.3 ± 6 kg	1.67 ± 0.6 m
Suchomel et al. [136]	Cross-sectional Observational	NCAA I soccer players	Tier 4	86 (12 female soccer players)	Familiarisation trials permitted	Yes	NS	63.0 ± 5.0 kg	1.66 ± 0.6 m
Szulc et al. [137]	Cross-sectional Observational	Youth and Senior National deaf soccer players	Tier 4	45 (20 deaf, 25 able hearing)	NS	NS	deaf = 23.7 ± 5.0 years hearing = 20.3 ± 3.8 years	deaf 61.2 ± 7.6 kg; hearing 60.3 ± 6.2 kg	deaf 1.65 ± 0.5 m; hearing 1.66 ± 0.5 m
Tallis et al. [138]	Cross-sectional Observational	Youth grassroot soccer players	Tier 2	30 (12 female)	Familiarisation trials permitted	NS	8.7 ± 1.3 years (pooled)	33.0 ± 2.8 kg (pooled)	1.40 ± 0.7 m (pooled)
Thomas et al. [139]	Cross-sectional Observational	2^nd^ Tier English soccer players	Tier 3	15	NS	Yes	20.6 ± 0.6 years	56.6 ± 6.3 kg	1.65 ± 0.7 m
Thomas et al. [140]	Cross-sectional Observational	Collegiate soccer players	Tier 3	11	< 7-days prior to testing	Yes	20.7 ± 0.8 years	54.70 ± 6.34 kg	1.66 ± 0.09 m
Thomas et al. [141]	Cross-sectional Observational	Semi-professional soccer players	Tier 2	14	NS	Sub-maximal trials	20.6 ± 0.6 years	56.2 ± 6.6 kg	1.65 ± 0.07 m

NS = not specified, NCAA, National Collegiate Athletic Association, M = mean, SD = standard deviation, kg = kilograms, m = meters, PHV = peak height velocity.

Pooled denotes that all participants included in the study are included for descriptive statistics.

McKay et al. [91] Performance Caliber Tier = sedentary (tier 0), recreationally active (tier 1), trained/ developmental (tier 2), highly trained/ national level (tier 3), elite/ international level (tier 4), world class (tier 5).

### 3.3. Force Plate Tests & Utility

**Fig 3:**
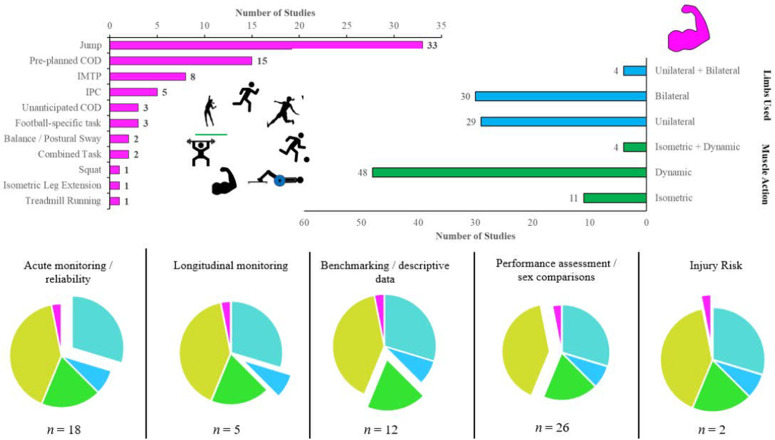
An overview of the force plate utility and tests.

The utility of force plate measures was categorised under the following terms and summarised in Fig 3:

- Acute monitoring/ reliability = studies that included single or repeated measures within seven days to assess within or test-retest reliability, or studies that included repeated measures within six weeks to assess a change in performance.

- Longitudinal monitoring = studies that included repeated measures over a period of six-weeks or longer (e.g., seasonal variations, monitoring through a mesocycle and/or competition)

- Benchmarking/ descriptive data = studies that report single measures and comparisons of different groups (e.g., ages, playing levels)

- Performance assessment/ sex comparisons = studies that assess the performance of a skills and/or compare sex differences in the same task (e.g., technique determinants)

Injury risk = studies that included comparisons of injured vs. non-injured players or prospectively followed injures.

The tasks measured by force plate tests ranged from jumping, change of direction, isometric tasks, balance, soccer simulation and running. Jumping was the most common test (bilateral CMJ = 25, unilateral CMJ = 7, drop jump = 6, bilateral squat jump = 5, unilateral squat jump = 1, CMJ-R = 2, broad jump = 1, depth jump with rebound = 1 hopping = 1, spike jump = 1) (Table 3). Pre-planned changes of direction were included in three change of direction angles (45º = 5, 60º = 2, 90º = 3, 180º = 5) with approach velocities ranging from 2.6 m·s ⁻ ¹ – 5.5 m·s ⁻ ¹ and approach distances ranging from 2 m – 15 m, or 50% of a pre-determined broad jump. Unanticipated changes of direction were included in three studies (~35–55º = 2, plus an unanticipated stop and jump task = 1 [125]) with approach velocities ranging from 3.3 m·s ⁻ ¹ – 3.5 m·s ⁻ ¹ and approach distances from 2 m or normalised to a maximum CMJ (i.e., to identify a relative maximal fall height). Isometric tasks included IMTP (*n* = 8), 90–90 isometric hamstring test (*n* = 5), 30–30 isometric hamstring test (supine with 30º of hip and knee flexion, *n* = 1), and isometric leg extension test (*n* = 1). A summary of the tasks measured by force plate tests are displayed in Figs 2-3.

**Table 3:** Force plate tests, model and setup.

Reference	Test	Number of trials, duration, and rest period (per limb)	Force Plate Model	Sampling Rate	Zeroing	Filters	Onset threshold	Instructions provided?	Additional analysis (i.e., integrated kinematics)
Abbott et al. [98]	IMTP (with straps)	3 trials 3-s effort 3-min rest	ForceDecks	1000 Hz	NS	NS	NS	Yes	No
Andersson et al. [99]	CMJ	3 trials Rest NS	AMTI	NS	NS	Low-pass filtered at 1050 Hz	NS	Yes	No
Barber et al. [23]	90−90 single leg isometric hamstring test (unilateral)	3 maximal trials 3–5-s duration rest not specified	Kistler 9286AA	1000Hz	NS	Raw data down sampled to 250 Hz. Only vertical force component analysed	5 SD of weighting period	Yes	No
Barnfield et al. [100]	Instep kicking action	1 rep per limb 5 variants 60-s rest between variants	NS	NS	NS	NS	NS	Yes	2D kinematics (estimated 3D)
Bishop et al. [101]	CMJ (unilateral, arms akimbo) SJ (unilateral, arms akimbo) DJ (unilateral, arms akimbo)	5 trial per limb SJ hold 2-s 30-s rest	AccuPower, AMTI	400 Hz	NS	NS	NS	Yes	No
Busko et al. [102]	CMJ (arms akimbo) CMJ (arm swing) Spike jump	3 trials 5-s for CMJ’s 60-s for spike jump	JBA Zb. Staniak	NS	NS	NS	NS	Yes	No
Butler et al. [103]	Stop jump (bilateral) Vertical jump to head a ball (bilateral, arm swing)	NS	AMTI	1080Hz	NS	NS	50Hz low pass filter	Yes	3D kinematics
Cabarkapa et al. [104]	CMJ (bilateral, arms akimbo)	3 trials 15-s rest	ForceDecks Max	1000 Hz	NS	20 N below system mass	NS	NS	No
Condello et al. [105]	Pre-planned 60º cut	5 trials per limb Approach speed between 3.5–5 m/s ^-1^ 2-min rest	Bertec Force Platform	2400 Hz	NS	NS	Force above 10 N for initial contact and continued until force dropped below 10 N	Yes	3D kinematics
Cortes et al. [106]	Anticipated side cutting task (~45º) Unanticipated side cutting task (~35–55º)	5 trials on DL only Approach speed > 3.5 m/s^-1^ 1-min rest	Bertec Force Plates, Model 4010	1080Hz	NS	Fourth-order Butterworth zero-lag filter 25 Hz cut-off frequency was used for vGRF	Force above 10 N for initial contact and continued until toe-off (toe-off threshold NS)	Yes	3D kinematics
Cortes et al. [107]	Pre-planned 45º cross-over step	4 trials (average) per timepoint (baseline and post fatiguing protocol) Fast as possible Rest NS	Bertec Force Platform	1200 Hz	NS	Fourth-order Butterworth zero-lag filter 25 Hz cut-off frequency was used for vGRF	Force above 10 N for initial contact and continued until toe-off (toe-off threshold NS)	NS	3D kinematics
Cuthbert et al. [108]	90−90 single leg isometric hamstring test (unilateral) 30−30 single leg isometric hamstring test (unilateral)	3 maximal trials 3–5 sec effort Rest NS	Kistler Type 9286AA	1000 Hz	NS	NS	NS	Yes	30 º kneeling IPC Eccentric hamstring strength
Della Villa et al. [109]	Pre-planned 60º side cut	3 trials per limb Fast as possible Rest NS	AMTI 400*600	120 Hz	NS	NS	NS	Yes	2D and 3D kinematics
Di Paolo et al. [110]	Single leg hop test (arm swing)	3 trials per limb >2-s hold Rest NS	AMTI 400*600	120 Hz	NS	NS	NS	Yes	2D and 3D kinematics
Di Paolo et al. [57]	Pre-planned 90º side cut	3 trials per limb Fast as possible	AMTI 400*600	120 Hz	NS	NS	NS	Yes	2D and 3D kinematics
Emmonds et al. [49]	IMTP (straps NS)	2 trials 5-s effort 3-5-min rest	AMTI, ACP,	1000 Hz	NS	NS	Onset of contraction = v.GRF > 40 N Vertical force–time curve was integrated over 100 and 300 ms windows from the onset of contraction	Yes	No
Emmonds et al. [97]	IMTP (straps NS)	2 trials 5-s effort 5-min rest	AMTI, ACP,	1000 Hz	NS	NS	5 SD of weighting period	Yes	No
Emmonds et al. [111]	CMJ (bilateral, arms akimbo) SJ (bilateral, arms akimbo) DJ (bilateral, arms akimbo) ISLE (hip angle = 110º, knee angle = 108.30 ± 2.31º)	3 maximal trials ISLE = 3-s Rest NS	Kistler 9287BA	1000 Hz	NS	NS	NS	Yes	No
Emmonds et al. [96]	IMTP (no straps)	2 trials 5-s effort 5-min rest	AMTI, ACP,	1000 Hz	NS	NS	NS	Yes	No
Emmonds et al. [43]	IMTP (straps NS)	2 trials 5-s effort 5-min rest	AMTI, ACP,	1000 Hz	NS	NS	NS	Yes	No
Fahey et al. [82]	CMJ (bilateral, arms akimbo) CMJ (unilateral, arms akimbo) CMJ-R (bilateral, arms akimbo)	3 trials Rest NS	Hawkin Dynamics Inc.	1000Hz	NS	Vertical force low pass filtered at 50 Hz	NS	Yes	No
Fahey et al. [84]	CMJ (bilateral, arms akimbo) CMJ (unilateral, arms akimbo) CMJ-R (bilateral, arms akimbo) IMTP (straps)	3 trials IMTP = 3–5-s	Hawkin Dynamics Inc.	1000Hz	NS	Vertical force low pass filtered at 50 Hz	Jumps = 5 SD of weighting period IMTP = 3 SD of weighting period	Yes	No
Fahey et al. [112]	CMJ (unilateral, arms akimbo)	3 trials per limb	Hawkin Dynamics Inc.	1000Hz	NS	Vertical force low pass filtered at 50 Hz	5 SD of weighting period	Yes	No
Fahey et al. [113] Muscles	CMJ (bilateral, arms akimbo) CMJ-R (bilateral, arms akimbo) IMTP (straps)	3 trials IMTP = 3–5-s effort Rest NS	Hawkin Dynamics Inc.	1000Hz	NS	Vertical force low pass filtered at 50 Hz	Jumps = 5 SD of weighting period IMTP = 3 SD of weighting period	CMJ – yes CMJ-R – yes IMTP – yes	No
Ferreria et al. [114]	SL CMJ (arm swing)	3 trials per limb ~30-s rest	ForceDecks, Vald Performance	1000 Hz	NS	None	NS	Yes	30m sprint
Fu et al. [115]	Single leg balance (arms crossed)	2 conditions 4 trials per limb 30-s effort 1-min rest	Kistler 9281B	40 Hz	NS	Reduced and smoothed to 20 Hz	NS	Yes	No
Gauffin et al. [94]	Half squat at 50% 1RM	6 trials Fast as possible Rest NA	PLA3–1D-7KN/ JBAZb	400Hz	NS	NS	NS	Yes	No
Goulart et al. [116]	Unanticipated stop-jump task or unanticipated 45º side cutting step task	5 successful trials Approach velocity must exceed 3.3m.s^-1^ Rest NS	Bertec Model 1060-NC	1080Hz	NS	NS	NS	NS	3D kinematics
Greska et al. [117]	30 cm DJ (arms swing NS)	3 trials Rest NS	Bertec, AM6110	600Hz	NS	NS	NS	Yes	3D kinematics
Harato et al. [118]	CMJ (bilateral, arms akimbo)	4-6 trials 45-60-s rest	AMTI, OR6-5-1	1000Hz	NS	Amplified (AMTI Model SGA6−3), digitized (DT 2801), and saved to dedicated computer software (Biojump)	NS	Yes	40m sprint
Haugen et al. [119]	CMJ	Minimum of 5 trials 45-60-s rest	AMTI, model OR6-5-1	1000 Hz	NS	Amplified (AMTI Model SGA6−3), digitized (DT 2801), and saved to dedicated computer software (Biojump)	NS	NS	No
Haugen et al. [120]	CMJ 20 kg loaded CMJ	3 trials 20-s rest	PASCO Passport	1000 Hz	NS	NS	NS	Yes	No
Ishida et al. [121]	CMJ (bilateral, polyvinyl chloride pipe across the back of the shoulders)	3 trials ≥ 30-s rest	PASPORT Force Platform	1000Hz	NS	NS	NS	Yes	
Ishida et al. [122]	Pre-planned 70–90º cut	Minimum of 6 acceptable trials	AMTI	1200Hz	NS	Butterworth low pass digital filter with cut-off frequencies of 25 Hz	Touchdown and take-off were defined as the instant that the vertical vGRF superseded and subsided past 20 N, respectively, for both penultimate and final contact. The weight-acceptance phase for both contacts was defined from touchdown to the point of maximum knee flexion	NS	3D kinematics
Jones et al. [31]	Pre-planned 90º cut	Minimum of 6 acceptable trials	AMTI, Model 600900	1200Hz	NS	Butterworth low pass digital filter with cut-off frequencies of 25 Hz	Weight acceptance phase of ground contact was defined as from the instant of initial contact (vGRF > 20 N) to the point of maximum knee flexion during ground contact as used previously	Yes	3D kinematics
Jones et al. [33]	Pre-planned 180º pivot	Minimum of 6 acceptable trials	AMTI, Model 600900	1200Hz	NS	Butterworth low pass digital filter with cut-off frequencies of 25 Hz	Weight acceptance phase of ground contact was defined as from the instant of initial contact (vGRF > 20 N) to the point of maximum knee flexion during ground contact as used previously	Yes	3D kinematics
Jones et al. [34]	Pre-planned 90º cut Pre-planned 180º pivot	Minimum of 10 trials 4 trials used for analyses	AMTI, Model 600900	1200Hz	NS	25 Hz Butterworth low pass digital filter	Weight acceptance phase of ground contact was defined as from the instant of initial contact (vGRF > 20 N) to the point of maximum knee flexion during ground contact as used previously End of contact was defined as the point when the vGRF subsided past 20 N for both penultimate and final contacts	Yes	3D kinematics
Jones et al. [32]	Pre-planned 180º pivot	Minimum of 6 acceptable trials	AMTI, Model 600900	1200Hz	NS	25 Hz Butterworth low pass digital filter	Weight acceptance phase of ground contact was defined as from the instant of initial contact (vGRF > 20 N) to the point of maximum knee flexion during ground contact as used previously End of contact was defined as the point when the vGRF subsided past 20 N for both penultimate and final contacts	Yes	3D kinematics Isokinetic dynamometry
Jones et al. [35]	Pre-planned and unplanned 45º sidestep and crossover step task	25 randomised trials: 5 pre-planned side-steps 5 pre-planned crossovers 5 unplanned side-steps 5 un-planned crossovers 1-min rest	Kistler 9286AA	1000Hz	NS	Fourth-order, zero-lag, 12-Hz low-pass Butterworth filter	The weight acceptance phase was defined from initial contact to the first minima of the resultant ground reaction force, and peak-push-off phase was defined from 10% from either side of the maximum resultant ground reaction force	Yes	3D kinematics
Khalid et al. [95]	CMJ (bilateral, without supporting arm swing) SJ (bilateral, without supporting arm swing)	3-trials per jump 1-min rest	Kistler 9286AA	1000 Hz	NS	NS	NS	Yes	Comparison between Optojump and Gyko inertial sensor system devise
Lesinski et al. [123]	CMJ (bilateral, arms akimbo) SJ (bilateral, arms akimbo) CMJ (unilateral, arms akimbo) SJ (unilateral, arms akimbo)	5 trials per test and limb 15-s rest	AccuPower, AMTI	4000 Hz	NS	NS	NS	Yes	No
Loturco et al. (106g)	CMJ (bilateral, on land, arms akimbo) CMJ (bilateral, on land, arms akimbo, 110% BW) CMJ (bilateral, on land, arms akimbo, 120% BW) CMJ (bilateral, on land, arms akimbo, 130% BW) CMJ (bilateral, water immersed, arms akimbo) CMJ (bilateral, water immersed, arms akimbo, 110% BW) CMJ (bilateral, water immersed, arms akimbo, 120% BW) CMJ (bilateral, water immersed, arms akimbo, 130% BW)	3 trails per jump and condition 2-min rest between jumps 5-min rest between conditions	AMTI Model OR6-WP	1000 Hz	NS	Fourth-order, low-pass Butterworth filter (20 Hz cut-off frequency	25 N	Yes	2D kinematics (sagittal plane)
Louder et al. [124]	Unanticipated single-leg land and hold Unanticipated single-leg land and side cut Unanticipated single-leg land and forward run	3-5 trials per condition Rest NS	AMTI	960 Hz	NS	Fourth-order, low-pass Butterworth filter (20 Hz cut-off frequency	NS	Yes	3D kinematics
Malloy et al. [125]	Kicking instep	As many as necessary until participant was satisfied of a maximal effort	AMTI	600 Hz	NS	NS	NS	Yes	2D kinematics (sagittal plane on plant leg only)
Orloff et al. [126]	Broad jump into pre-planned 45º cut (arm swing NS)	3 trials Rest NS	Kistler	1000 Hz	NS	Low-pass filter (10 Hz)	NS	Yes	2D kinematics EMG
Rath et al. [127]	90–90° single leg isometric hamstring test (unilateral)	3 trials per limb 3-5s effort 1-min rest	Kistler Type 9286AA	1000 Hz	NS	Raw force time data was down sampled to 500-, 250-, and 100 Hz	5 SD of weighting period	Yes	No
Ripley et al. [68]	90–90° single leg isometric hamstring test (unilateral)	3 trials per limb 3-5s effort Rest NS	Kistler Type 9286AA	1000 Hz	NS	NS	5 SD of weighting period	Yes	No
Ripley et al. [46]	90–90° single leg isometric hamstring test (unilateral)	3 trials per limb 3-5s effort 1-min rest	Kistler Type 9286AA	1000 Hz	NS	No filters. Raw force time data was down sampled to 250 Hz	5 SD of weighting period	Yes	No
Ripley et al. [25]	CMJ (bilateral, arm swing NS)	2-3 trials Rest NS	Hawkin Dynamics	1000 Hz	NS	NS	30 ms prior to vGRF exceeding 5 SD of weighting period	Yes	No
Romero-Moraleda et al. [128]	CMJ (bilateral, arms akimbo)		Force-Decks FD4000	1000 Hz	NS	NS	A drop of 20 N from baseline force (recorded during the weighing phase) was observed	Yes	No
Romero-Moraleda et al. [129]	CMJ (bilateral, arms akimbo) IMTP (no straps)	2-3 trials 2-min rest between trials and exercises	ForceDecks	NS	NS	NS	NS	Yes	No
Seraphin et al. [130]	CMJ (bilateral, arms akimbo)	3 trials Rest NS	Kistler 9290DD	500 Hz	Yes	Filter smoothing with a moving average of 0.005 s	NS	Yes	No
Siegel et al. [131]	Incremental treadmill running test.	Until exhaustion	Bertec	1080 Hz	NS	7^th^ order, zero-lag Butterworth low pass filter with a cut of frequency of 65 Hz after the first 10 steps	Foot contacts were identified when vGRF exceeded 100 N	No	No
Sharma & Singh [132]	31 cm DJ (bilateral, arms swing)	Repeated trials until 3 consecutive fails Rest NS	AMTI	1200 Hz	NS	4^th^ order, zero-lag Butterworth low pass filter with a cut of frequency of 65 Hz after the first 10 steps	NS	Yes	3D kinematics
Smith et al. [133]	Depth jumps with rebound	0, 3, 6, 9 or 12 reps 10-s rest	AMTI	1000 Hz	NS	NS	NS	Yes	No
Steig et al. [134]	CMJ (bilateral, arm swing) 15 cm DJ (bilateral, arms akimbo) 30 cm DJ (bilateral, arms akimbo) 45 cm DJ (bilateral, arms akimbo) 60 cm DJ (bilateral, arms akimbo)	3 trials per jump Rest NS	Kistler 9281B13	250 Hz	NS	NS	NS	NS	Isokinetic dynamometry
Struzik & Pietraszewski [135]	CMJ (bilateral, polyvinyl chloride pipe across the back of the shoulders) SJ (bilateral, polyvinyl chloride pipe across the back of the shoulders)	2 trials per test 30-s rest	Rice Lake Weighing Systems	1000 Hz	NS	Low-pass Butterworth filter with a cutoff frequency of 10 Hz	5 N	Yes	No
Suchomel et al. [136]	CMJ (bilateral, arms akimbo) CMJ (bilateral arm swing) Spike jump (arm swing)	6-s rest between CMJ and CMJ arm swing trials 1-min rest between spike jump trials 3-min rest between jump variants	“JBA” Zb. Staniak	NS	NS	NS	NS	Yes	Isokinetic dynamometry
Szulc et al. [137]	CMJ (bilateral, arms akimbo)	3 trials 1-min rest	PASCO, Pasport PS-2142	1000 Hz	NS	Raw unfiltered data used for analysis	Onset threshold NS 10 N to determine take-off and landing	Yes	Comparison between My Jump 2 App for time in air and take off velocity method
Tallis et al. [138]	Pre-planned modified 505 test	Minimum of 6 trials per limb Rest NS	AMTI 600,900	1200 Hz	NS	Butterworth low pass digital filter with cut-off frequency of 25 Hz,	vGRF > 20 N for initial contact vGRF < 20 N for both penultimate and final contact	Yes	3D kinematics
Thomas et al. [139]	CMJ (bilateral, arms akimbo) IMTP (no straps)	3 trials 1-min rest	Kistler 9286AA	1000 Hz	NS	Raw unfiltered data used for analysis	5 SD of weighting period	Yes	No
Thomas et al. [140]	180° turns	6 successful trials 2-min rest	AMTI (600900)	1200 Hz	NS	Butterworth low pass digital filter with cut-off frequency 25 Hz	vGRF > 20 N for initial contact vGRF < 20 N for both penultimate and final contact	Yes	3D kinematics

CMJ = countermovement jumps, SJ = squat jump, DJ = drop jumps, SD = standard deviation, vGRF = vertical ground reaction force, NS = not specified, IMTP = isometric mid-thigh pull, -s = seconds, -min = minute(s), ms = milliseconds, SD = standard deviations

### 3.4. Metrics

Peak force was the most common metric, reported in 34 studies with three of those studies reporting net peak force [108,113,84]. This was followed by joint moments which was reported by twenty-four studies who used integrated kinematics alongside force plate measures. Four studies reported rate of force development (RFD) including average RFD over 100 ms [46,68], average RFD from 0–200 ms [46,68], propulsive RFD [49] and maximum concentric RFD [115], noting that concentric refers to the propulsion phase. Four studies reported force at 100 ms [23,25,46,68] and 200 ms [23,25,46,68], and one study reported impulse and 100 ms and 300 ms [49]. Twenty-six studies reported jump height which was calculated using either impulse-momentum theorem [,^82^, ^84^, ^99^, ^101^, ^102^, ^104^, ^112^,^113^,^120^,^119^,^128^,^129^,^132^,^137^,^140^] or flight time [111,115,132,135,136], with five studies not specifying the method used to calculate jump height [121,122,142,130,134]. Inter-limb asymmetry was reported in seven studies [101,104,108,115,142,130,131]. Eleven studies included the ‘best trial’ for analysis [43, 49, 96, 97, 99, 102, 111–120] whilst the remaining studies included the ‘average of trials’ for analysis. Thomas et al. [140] reported the best trial for the IMTP but average of trials for CMJ metrics and was the only study to include both.

### 3.5. Mixed methods appraisal tool assessment

The results of the mixed methods appraisal tool for each study are presented in Table 4. All studies provided clear research questions designs that enabled the data collected to answer the research question. Thirteen studies were scored as quantitative non-randomized, and forty-four studies were scored as quantitative descriptive.

**Table 4:** Study scores from the MMAT [93].

	All studies		Quantitative non-randomized					Quantitative descriptive				
Study	1	2	1a	2a	3a	4a	5a	1b	2b	3b	4b	5b
Abbott et al. [98]	Y	Y	Y	Y	Y	Y	Y					
Andersson et al. [99]	Y	Y	Y	Y	Y	C	Y					
Cortes et al. [106]	Y	Y	Y	Y	Y	C	Y					
Di Paolo et al. [57]	Y	Y	Y	Y	Y	Y	Y					
Emmonds et al. [111]	Y	Y	Y	Y	Y	Y	Y					
Fahey et al. [113]	Y	Y	Y	Y	Y	Y	Y					
Fahey et al. [84]	Y	Y	Y	Y	Y	Y	Y					
Goulart et al. [94]	Y	Y	Y	Y	Y	N	Y					
Greska et al. [71]	Y	Y	Y	Y	Y	Y	Y					
Ishida et al. [121]	Y	Y	Y	Y	Y	C	Y					
Ishida et al. [122]	Y	Y	Y	Y	Y	Y	Y					
Khalid et al. [95]	Y	Y	Y	Y	Y	Y	Y					
Romero-Moraleda et al. [128]	Y	Y	Y	Y	Y	Y	Y					
Romero-Moraleda et al. [129]	Y	Y	Y	Y	Y	Y	Y					
Steig et al. [131]	Y	Y	Y	Y	Y	Y	Y					
Barber et al. [23]	Y	Y						C	Y	Y	Y	Y
Barnfield et al. [100]	Y	Y						C	C	Y	Y	Y
Bishop et al. [101]	Y	Y						C	Y	Y	Y	Y
Busko et al. [102]	Y	Y						C	Y	Y	Y	Y
Butler et al. [103]	Y	Y						Y	Y	Y	Y	Y
Cabarkapa et al. [104]	Y	Y						Y	Y	Y	Y	Y
Condello et al. [105]	Y	Y						Y	Y	Y	Y	Y
Cortes et al. [143]	Y	Y						C	Y	Y	Y	Y
Cuthbert et al. [144]	Y	Y						C	Y	Y	N	Y
Della Villa et al. [75]	Y	Y						Y	Y	Y	Y	Y
Di Paolo et al. [109]	Y	Y						Y	Y	Y	Y	C
Emmonds et al. [17]	Y	Y						C	Y	Y	Y	Y
Emmonds et al. [49]	Y	Y						C	Y	Y	Y	Y
Emmonds et al. [43]	Y	Y						C	Y	Y	Y	Y
Emmonds et al. [96]	Y	Y						C	Y	Y	Y	Y
Fahey et al. [112]	Y	Y						C	Y	Y	Y	Y
Fahey et al. [145]	Y	Y						Y	Y	Y	Y	Y
Fu et al. (2023)	Y	Y						Y	Y	Y	Y	Y
Gauffin et al. [94]	Y	Y						C	C	Y	Y	C
Harato et al. [118]	Y	Y						C	Y	Y	Y	Y
Haugen et al. [119]	Y	Y						Y	Y	Y	N	C
Haugen et al. [120]	Y	Y						Y	Y	Y	Y	Y
Jones et al. [34]	Y	Y						Y	Y	C	Y	Y
Jones et al. [33]	Y	Y						Y	Y	C	Y	Y
Jones et al. [32]	Y	Y						Y	Y	C	Y	Y
Jones et al. [35]	Y	Y						Y	Y	C	Y	Y
Jones et al. [31]	Y	Y						Y	Y	C	Y	Y
Lesinski et al. [123]	Y	Y						C	Y	Y	Y	Y
Loturco et al. [142]	Y	Y						C	Y	Y	Y	Y
Louder et al. [124]	Y	Y						C	Y	Y	Y	Y
Malloy et al. [125]	Y	Y						Y	Y	Y	Y	Y
Orloff et al. [126]	Y	Y						C	Y	Y	Y	Y
Rath et al. [127]	Y	Y						C	Y	Y	Y	C
Ripley et al. [46]	Y	Y						Y	Y	Y	Y	Y
Ripley et al. [68]	Y	Y						Y	Y	Y	Y	Y
Ripley et al. [25]	Y	Y						Y	Y	Y	Y	Y
Seraphin et al. [130]	Y	N						C	Y	C	Y	C
Sharma & Singh [132]	Y	Y						Y	Y	Y	Y	Y
Siegel et al. [131]	Y	Y						C	Y	Y	Y	Y
Smith et al. [133]	Y	Y						C	Y	Y	Y	Y
Struzik & Pietraszewski [135]	Y	Y						C	Y	Y	Y	Y
Suchomel et al. [136]	Y	Y						C	Y	Y	Y	Y
Szulc et al. [137]	Y	Y						C	Y	Y	Y	Y
Tallis et al. [138]	Y	Y						C	Y	Y	Y	Y
Thomas et al. [139]	Y	Y						C	Y	Y	Y	Y
Thomas et al. [140]	Y	Y						C	Y	Y	Y	Y
Thomas et al. [141]	Y	Y						C	Y	Y	Y	Y

1 = Are there clear research questions? 2 = Do the collected data allow the research question to be answered?

1a = Are the participants representative of the target population? 2a = Are measurements appropriate regarding both the outcome and intervention (or exposure)? 3a = Are there complete outcome data? 4a = Are the confounders accounted for in the design and analysis? 5a = During the study period, is the intervention administered (or exposure occurred) as intended?

1b = Is the sampling strategy relevant to address the research question? 2b = Is the sample representative of the target population? 3b = Are the measurements appropriate? 4b = Is the risk of non-response bias low? 5b = Is the statistical analysis appropriate to answer the research question?

Scoring: Y = Yes, N = No, C = Can’t tell.

## 4. Discussion

### 4.1. Overview

As participation rates in female soccer continue to increase [5,6], research in female soccer has predominantly focused on injury incidence and strength and conditioning practices at senior level. However, some researchers have highlighted the need for a critical evaluation of the existing research and the production of higher-quality research questions, designs and outputs [9,10,146]. In particularly concerning recommendations are made to support female health and performance [19], and for initiatives promoting youth female participation in school soccer [147]. This scoping review identified 63 studies which assessed force in female soccer players using force plates, with fifty of these studies being published in the last decade, highlighting an increasing trend of force plate information across different performance levels and age. Since the first publication of force plate assessment with female soccer players in 1993, the number of publications per year has steadily increased since then, with 2025 showing the largest number of publications to date. To the best of our knowledge, this is first scoping review highlighting current research using force plates in female soccer and identifying gaps within the literature which may require further investigation. The findings of this scoping review highlight the majority of research including force plates in female soccer players is focused on performance assessments/ sex comparison on senior elite players with jumping tasks, in particular the bilateral CMJ being the most frequently utilised. These findings align with previous researchers who reported a bias towards senior and elite female soccer players [37]. Based on these results there is a clear need for more research to emphasise on youth players, detailed analysis of force-time characteristics, with integration of additional sports technology (i.e., motion capture) in applied settings, collection and analysis of multi-centre studies, the development of ‘best practice’ for unilateral and CMJ-R tests, and maximal and rapid isometric force assessment and whether this relates to prospective or retrospective injury.

### 4.2. Study demographics

Forty-three studies (68%) investigated senior female soccer players (≥18 years of age), whereas nine (14%) investigated youth soccer plyers (≤18 years of age). Eleven studies included both youth and senior players (17%). Sample size ranged from six [94,95] to 157 [96,97,35, 95, 111, 148]. Nineteen studies (30%) conducted *a*-priori sample size estimation to determine sample size requirements [11,20,28,40, 47,83,92,94,105,120,146,142, 126], with Abbott et al. [98] conducting a sensitivity analysis based on a sample size of 16, noting that they were limited to data collection within a single soccer club. This is a potential limitation for practitioners and researchers who should consider if there are sufficient participants involved in the study to be adequately powered before drawing conclusions from the reported results. Insufficient sample sizes are likely to violate normality assumptions with confidence intervals considered too large for realistic interpretation and application of their findings [149]. However, alternative statistical approaches (i.e., Bayesian) may provide a better probabilistic approach with simplistic statements for interpretation especially in studies with low sample sizes [150]. The results of this review are biased towards teams competing in Europe followed by the United States of America. As practitioners and researchers may be constrained to a single club/centre, which may reflect one style of practice within a club/centre, different sporting contexts (i.e., training and competition schedules) and levels of performance, future researchers should strive to complete data collection across multiple clubs/centres to include a diverse range of data in a specific population (e.g., youth, senior, recreational, elite, international soccer players). The performance calibre according to McKay et al. [91] ranged from tier 2 (trained/developmental) [103,109,110] through to tier 5 (world class) [128]. One study included in this review [94], did not provide sufficient information to classify the performance calibre of the participants, which is described as not specified in Table 2. Tier 4 (elite/international) participants were the most investigated (*n* = 26, 41%) of all studies which included both senior and youth soccer players, which is in agreement with Okholm Kryger et al. [10] who identified 51% of studies (*n* = 189) included elite players. Researchers should design studies based on appropriate sample size estimations, which is a frequent requirement in peer-reviewed journals ensuring transparency for readers who could have confidence when interpreting results [151,149]. Multi club/centre studies could also provide clearer information to better understand the health (e.g., injury risk) and performance (e.g., physical qualities) of female soccer players including the responsible mechanisms [152,147,19].

### 4.3. Force plate tests

Twenty-nine studies investigated unilateral tasks (e.g., unilateral jumps, changes of direction and 90−90 test), thirty investigated bilateral tasks (e.g., CMJ, CMJ-R, IMTP) and four studies included both (e.g., bilateral and unilateral CMJ). Dynamic tasks (*n* = 47) were more common than isometric tasks (*n* = 11), with four studies including a combination of dynamic and isometric tasks. Jumping was the most common task (*n* = 33), with the bilateral CMJ being the most investigated (*n* = 23) which are in agreement with the findings reported by Badby et al. [37] and Guthrie et al. [38] across different team sports. Twenty-four of these studies were conducted in senior soccer players, five studies included youth players, while three studies included both youth and senior players [113,82,119] (Table 2). Pre-planned change of direction was the second most investigated task (*n* = 15), with angles ranging from 45º [106,107,95,127], 60−90º [105,109,57,31–33], and 180º [32,34,35,139,153]. Approach velocities ranged from 2.6 m·s ⁻ ¹ – 5.5 m·s ⁻ ¹ and approach distances ranging from 2 m – 15 m, or 50% of a pre-determined broad jump. All of the studies that investigated pre-planned change of direction were laboratory based using either two-dimensional (2D) or 3D kinematics and additional biomechanical assessment such as muscle activity through surface electromyography [127] and isokinetic dynamometry [31,35,135,137]. Whilst this provides valuable information to practitioners relating to kinetics, kinematics and muscle activity, this is often time consuming and expensive, meaning that applied practitioners may not have the option to involve this in their assessment of large groups of players, especially during in-season competition. Recently, the field-based Cutting Movement Assessment Score [154] has been proposed as a tool that could provide practitioners with valuable information alongside force plate assessment without time restrictions or additional cost. The most common isometric test was the IMTP, however, there was methodological variability across the identified studies. Six studies did not report the use of weightlifting straps [49,43, 96,97,130,140] whilst only three studies reported the use of weightlifting straps [98,113,84], despite standardised guidelines on the implementation of the IMTP [75]. By not utilising weightlifting straps, researchers and practitioners are unable to confidently assess and interpret maximal and rapid lower limb isometric force production, as values may be limited by grip strength [155,156]. Senior players were recruited for three studies investigating the IMTP [98,111,130], with sample sizes ranging from 10 [111] to 28 [130], but only Abbott et al. [98] conducting a power calculation for sample size. Studies including senior players were single club samples, whereas Emmonds et al. [43,49, 96-97] provided IMTP data from three elite English soccer academies, thus highlighting a gap in the knowledge for senior players from multiple clubs/centres. This provides practitioners with a more accurate representation of the maximal force production derived from the IMTP for players competing at the same level, especially as single club studies may reflect the training practices of a single club rather than the wider performance level. Isometric hamstring force assessments was the second most common isometric test in this review and included the 90:90 test [23,108,25,46,68] and the 30−30 test [108], with all studies conducted in senior players and no data provided for youth players. As hamstring strain injuries are a primary concern for female players akin to male players [157,158,159], and the number of high intensity actions during match play increase with increasing age [7,89] and competition [8], isometric hamstring force assessment can be valuable for practitioners to prioritise training goals and profile injury risk given its role in sprint performance [160]. Currently there are no benchmarks for female soccer players for isometric (i.e., IMTP, 90−90 or 30−30 tests) or dynamics tasks (i.e., CMJ, CMJ-R) at different levels of performance (e.g., youth, senior and international standards). This would offer practitioners valuable information for strength and conditioning program design, monitoring long-term athletic development and injury risk profiling [161,9,10]. Therefore, future researchers should consider developing benchmarks for these qualities including peak and rapid force production during isometric tests, and phase-specific qualities to better understand the mechanisms responsible for superior jump performance.

### 4.4. Force Plate Utility

The utility of force plates in this review included: acute monitoring/ reliability (*n* = 18), longitudinal monitoring (*n* = 5), benchmarking/ normative data (*n* = 12), performance assessment/ sex comparisons (*n* = 26), and injury risk (*n* = 2) (Fig 3). Studies designed for acute monitoring/ reliability was mostly conducted with senior players [98,99,107,114,116,121,122], or a combination of youth and senior players [23,108,113,25,46,68]. Six studies reported within session reliability [99,23,101,25,68,138] and seven studies reported test-retest reliability [108,112,82,116,121,122,46]. Jumping was the most common task for monitoring studies, namely the bilateral CMJ [99,101,82,120,121,122,123,138], and isometric hamstring assessments, namely the 90–90 test [23,108,25,46,68]. Acute fatigue monitoring was investigated following competitive match play [98,99,112,116,121,122] or following a functional agility fatigue protocol [107]. Five studies included longitudinal monitoring, three of which investigated senior players [117,128,129] and two studies monitored youth players [43,84]. In youth players, Fahey et al. [112] monitored unilateral CMJ over a six week strength and plyometric training mesocycle to assess whether test-retest reliability improved and determine if force-time characteristics changed in the CMJ (bilateral and unilateral), CMJ-R and IMTP. Meanwhile, Emmonds et al. [43] reported seasonal changes in the IMTP gross peak force in youth players from three English academies, and Greska et al. [117] observed the effect of a 10-week feedback inclusive neuromuscular intervention on isometric strength (which was not assessed by force plates), jump performance and landing mechanics (with integrated kinematics) in senior players. The results of these studies demonstrate that practitioners can effectively use force plates to reliably monitor changes in force-time characteristics during jumping and isometric assessments. However longer study durations (i.e., over multiple seasons) with more testing points will provide practitioners information on the fluctuations of force-time characteristics and the mechanisms responsible for these changes in female soccer players (both youth and senior). Only one study monitored changes in CMJ performance through an eumenorrheic menstrual cycle in elite female soccer players [129]. The same authors also monitored CMJ performance of elite Spanish female soccer players during the FIFA Women’s 2023 World Cup [128]. This is the only study in this review to include tier 5 (World Class) players and provides valuable information for practitioners working with international players. Further research in this population is needed especially during competitions as the physical demands are greater at international standards compared to domestic [8]. Twelve studies provided descriptive data with jumping being the most common test, in particular the bilateral CMJ [111,119,132,135,136,137]. Other jump tests include unilateral CMJ [101,115], squat jump [111,136] and drop jump [101,111]. Drop jump heights ranged from 30 cm to 60 cm, however, actual fall heights can vary between players when using the same height box with variability >20% [144]. This highlights the importance of tracking fall height in order to ensure consistency between athletes and tests and inform interpretation. The CMJ-R is an alternative test that allows players to fall from their maximum CMJ height before executing the rebound jump, thus providing the same relative intensity. The CMJ-R allows the assessment of ballistic (i.e., slow stretch shortening cycle) and plyometric ability (i.e., fast strength shortening cycle) [162–164], although the validity has been questioned [162]. Within female soccer players, the CMJ-R has demonstrated good test-retest reliability [82,162], which remains stable during in-season strength training in youth female soccer players [84]. However, there is no benchmarking data for CMJ-R in female soccer players, especially in senior and international players. Future research is also needed for better standardisation and coaching of fast stretch shortening cycle. This would allow for clear comparisons to be made between studies and accurate interpretations so that coaches to appropriately design strength and conditioning program [165]. Descriptive data for the IMTP has been reported in youth players according to chronological age [49,97] and maturation status [49,96] across multiple English soccer academies. Only one study included within this review provided descriptive data on vertical ground reaction force during a unilateral balance task [94], but this only included a sample size of six players who were not identified to a specific performance calibre [91]. Performance assessment/ sex comparison studies were the most common utility of force plates in this review, with change of direction being the most common test for this comparison [105,106,109,31–35,95,127,141,139] and one study investigating kinematics and kinetics of an in-step pass [100]. Jumping was the second most common test for performance assessment/ sex comparison [102,113,118,142,124,133,134,140], however Thomas et al. [140] is the only study to compared force-time curves of strength-matched male and female soccer players using the IMTP. Fahey et al. [113] compared force-time characteristics of the bilateral CMJ and CMJ-R between stronger and weaker players, however without benchmarks for IMTP in female soccer players the groups were based on a top versus bottom quartile split. Future researchers should aim to develop phase-specific benchmarks for force-time characteristics in the CMJ, CMJ-R and IMTP for both youth and senior players. Two studies included in this review focused on injury risk, comparing ACL injured players and healthy controls [104,57]. Cabarkapa et al. [104] assessed bilateral CMJ with three-dimensional analysis for injured players 11–13 months post-surgical repair, whereas Di Paolo et al. [57] prospectively followed first-to-third division Italian soccer female soccer players using two- and three dimensional analysis of a pre-planned 90º change of direction. It is surprising that only one study published data on prospective injury risk in female soccer players, especially as injury risk and incidence is greater in females compared to males [12,13], which spans both youth [14] and senior players [12–14]. This is likely do the complexity and challenges faced by practitioners and researchers when attempting to conduct prospective injury risk studies [166]. In summary, the utility of force plates is mostly for performance assessment/ sex comparisons, including change of direction and jump performance. Other utilities include acute monitoring/ reliability, longitudinal monitoring, benchmarking and descriptive data, and injury risk. There are current gaps within the literature with a lack of benchmarking data for youth and senior players across different phases (e.g., braking, propulsion) of various jump tasks (e.g., CMJ, CMJ-R) and isometric tasks including peak and rapid force production (e.g., force at 200 ms). Further research is also needed on international players to better understand the physical qualities of these players with the increasing physical match demands with age [7,89] and competition [8]. Benchmarks of these qualities would provide practitioners with the ability to profile performance, and injury risk and is required for prospective monitoring.

### 4.5. Metrics

Peak force was the most common metric and reported by 34 studies across different dynamic tasks (e.g., jumping, change of direction, landing) and isometric tasks (e.g., IMTP, 90−90 test). There were inconsistencies in the included studies for reporting peak force (i.e., gross or net) which makes comparisons between studies difficult. Gross peak force (i.e., peak force including body or limb weight) was reported in seven studies for the IMTP [98,49,43, 96,97,84,140], 90−90 and 30−30 hamstring assessments [108], as well as during different phases of jumping tasks such as braking and propulsive [104,115]. Three studies reported net peak force (i.e., peak force minus body or limb weight) during the IMTP [113,84,140]. Net peak force was originally proposed by Haff et al. [155,156], removing the influence of body mass because gross peak force will inflate peak force values and would increase any differences observed between groups if one has a higher body mass, as those observed by Emmonds et al. [49,43, 96,97]. With inconsistencies in the included studies for reporting peak force (i.e., gross or net), practitioners and researchers who report gross force are encouraged to include body mass for context, especially when monitoring youth players [72,75,155,156], which enables accurate interpretation of force production capability. Sixteen studies ratio scaled peak force to body mass [98,105,110,49,43, 96,97,113,84,121,95,126,139–140,141], but only Fahey et al. [113,84] and Thomas et al. [140] reported ratio scaled net peak force. Ratio scaling peak force to body mass allows practitioners and researchers to make comparisons between players, performance levels and ages across different studies, however practitioners and researchers should ensure that the same standard operate procedures have been adhered to, including the calculation of peak force (i.e., gross or net peak force) as gross force will inflate peak force values and create greater differences between groups, especially if one group has a greater body mass (e.g., youth vs senior players). Butler et al. [103], Steig et al. [134] and Sharma et al. [132] expressed peak vertical ground reaction force as a percentage of body mass, however practitioners should include body mass alongside this for further context. It is also important to note that rapid force expression is also crucial for female soccer players, as powerful actions often precede a goal scoring opportunity [167]. Rapid force metrics ranged from force at early timepoints (e.g., force at 100 ms and 200 ms) [23,25,46,68], RFD at early timepoints (e.g., RFD 0−100 ms, RFD 0−200 ms) [46,68], and RFD during specific-phases of jump tasks (i.e., propulsive RFD) [104,111,115]. Two studies also reported force at early timepoints normalised to peak force (e.g., force at 100 ms divided by gross peak force) to develop a quadrant style report for practitioners to identify training priorities (e.g., peak force development, rapid force development, or a combination). Rapid force metrics reported from the 90−90 test, demonstrated moderate to good reliability [23,25,46], with moderate to excellent reliability reported with lower sampling rates [68]. Emmonds et al. [49] reported impulse at 100 ms and 300 ms during the IMTP but as impulse is the product of ∆*force x* ∆*time*, it would appear redundant as the time component is fixed. Future research is needed to identify benchmarks for peak and rapid force metrics across a range of tests (e.g., IMTP and 90−90) for female soccer players.

Twenty-six studies reported jump height which was calculated using either impulse-momentum theorem [99,101,102,104,82, 84, 112,113,120,119,128,129,132,137,140] or flight time [111,115,132,135,136], with five studies not specifying the method used to calculate jump height [121,122,142,130,134]. Previous authors have confirmed greater accuracy when calculating jump height using the impulse-momentum method compared the flight time, which can overestimate jump height [168,145,169,170], with changes in flight position prolonging their flight time and leading to the overestimation of jump height. Terminology for the different phases of jump tasks were inconsistent with some authors describing phases as eccentric and concentric [104,115,129], whereas others describe the different phases as braking and propulsive [82, 84, 112,113,128,140], as outlined by McMahon et al. [41]. Braking and propulsive are more appropriate and refer to biomechanical actions, whereas eccentric and concentric describe to muscle actions [42]. Five studies measured both the CMJ and squat jump [101,111,123,142,136], but only Suchomel et al. [136] reported eccentric utilization ratio (i.e., CMJ height ÷ squat jump height). Other ratio metrics identified in this review include reactive strength index [111,113,82,136] and reactive strength index modified [82, 84, 112,113]. Ishida et al. [121] reported reactive strength index, however this was calculated as jump height divided by time to take-off, which is in fact the calculation for reactive strength index modified [171]. Interestingly, not all authors reporting ratio metrics reported the constituent parts (e.g., reactive strength index = jump height divided by ground contact time), which is important for practitioners, as this explains the mechanisms responsible for changes in such scores. Notably, authors reported mean force during jumping tasks [104,82, 84, 112–113,129,140], which have demonstrated greater sensitivity for monitoring acute fatigue [143], especially as peak force values represent 1 ms of the data when collected at 1000 Hz and could be affected by noise [172]. Alongside jump height, peak power was often reported (either as gross or relative to body mass). Practitioners are reminded that power is a compound variable calculated as the product of instantaneous ground reaction force and instantaneous velocity, and in not reflective physiological power generated by the athletes muscles, nor jumping performance [21]. Fifty-two studies included the average of trials for analysis, whereas 10 studies included the best trial (i.e., the best score from multiple trials) [98,99,49,43, 96–97, 120,142,124]. Thomas et al. [140] reported the best trial for the IMTP but average score for CMJ metrics and was the only study to include both. Previous authors have reported greater reliability during jump tasks by taking the average of two to three trials compared to the best score [148,173]. The authors did recommend that practitioners conduct reliability of their own data to ensure the noise within the data collection and associated error (i.e., natural variation) are sufficient to identify thresholds for detecting a meaningful change. However, future research is needed to establish whether the average or best trial is most appropriate in female soccer players for monitoring and benchmarking purposes. In summary, peak force was the most popular metric reported but gross or net peak force was inconsistently used, however future research should establish benchmarks for specific tests along with rapid force assessment to identify training priorities.

### 4.6. Limitations and recommendations for future research

A limitation of the current review is that the majority of research is targeted towards senior and elite soccer players, which is in agreement with Okholm Kryger et al. [10]. The same authors suggested that existing research currently focuses on injuries in female soccer and emphasises descriptive data of injuries. Therefore, future researchers should identify injury mechanisms, risk factors and inform program design to enhance performance and reduce injury risk at all levels and ages. Randell et al. [19] concluded that high quality research is needed to inform interventions that support the health and performance of female players. However, benchmarks of physical qualities are needed at different stages of age and performance level to identify potential injury risk factors, especially given that strength is a well-established modifiable risk factor, in particular knee injuries [174]. This information would enable practitioners to design programs to prepare players looking to transition from youth into senior ages, or domestic to international competition levels. A second limitation of the existing research is that these often include a single club which may reflect a single style of practice. Emmonds et al. [49,43,96] reported descriptive data from three elite English soccer academies, however no multi-club study has been conducted at senior levels. Badby et al. [175] reported normative and objective data for the CMJ, CMJ-R and IMTP from seven male English youth academies, however this information may not accurately inform the needs of the female soccer players [17], thus future research should aim to replicate this design in females. Only a small number of studies were identified within this review that specified whether an appropriate power calculation was used to determine a sufficient sample size for the intended research question. This is a limitation for practitioners and researchers who should consider if there are sufficient participants involved in the study to be sufficiently powered before drawing conclusions from the reported results [149]. Inconsistencies were also observed in the included studies for reporting peak force and whether gross (i.e., peak force including body or limb weight) or net peak force (i.e., peak force minus bodyweight) were used. Practitioners and researchers should clearly report whether gross or net peak force are included so that comparisons between studies can be made. If gross peak force is reported then body mass should be included because gross peak force will inflate peak force values and would increase any differences observed between groups if one has a higher body mass. The recommendations for future research are outlined in Table 5.

**Table 5:** Recommendations future force plate research in female soccer.

Force Plate Utility	Future Research Recommendations
Acute monitoring	Time course of fatigue and acute fatigue monitoring of phase-specific variables for jump tasks (e.g., CMJ, CMJ-R) and force-time characteristics for isometric assessment. Monitoring fatigue and changes in performance of peak and rapid force measures during international tournaments.
Longitudinal monitoring	Monitoring changes in force-time characteristics during maturation and age.
Benchmarking/ descriptive data	Phase-specific benchmarks for dynamic tasks (e.g., CMJ, CMJ-R) Benchmarks for peak and rapid force production during isometric tasks (i.e., IMTP, 90−90 test) Development of multi-club studies to aid benchmarking collation and observational differences.
Performance assessment/ sex comparisons	Force assessment comparison of peak and rapid force production between youth and senior players and between domestic and international players.
Injury risk	Assessment of peak and rapid force production and associations with prospective injury. Longitudinal monitoring of force assessment with prospective injury (e.g., changes during maturation into senior soccer)

CMJ = countermovement jump, CMJ-R = countermovement rebound jump, IMTP = isometric mid-thigh pull

## 5. Conclusions

The present scoping review highlights multiple gaps within the literature that should be addressed to support the health and performance of the female soccer players [18,19]. Previous researchers have identified a bias of research in female soccer towards senior, elite players. The most investigated topics include injury and strength and conditioning [10], with performance analysis (physical and technical) and talent identification/ growth and maturation as other common themes [152]. The findings of this scoping review highlight that the majority of researchers using force plates in female soccer is targeted towards elite and senior players using jumping activities followed by pre-planned change of direction and isometric tasks (e.g., IMTP, 90−90 test). Peak force was the most reported metric, however gross or net peak force were inconsistently used between studies and there was a gap in the research using rapid force assessment (e.g., force at early timepoints) with female soccer players which would also offer practitioners with valuable information to identify training priorities. The most common utility for force plate assessments was performance assessment/ sex comparisons, however Emmonds et al. [17] and Beato et al. [176] have proposed that more research is needed to verify the reliability of different tests and metrics and establish female soccer specific normative data at youth and senior levels, as comparison to males is not applicable. Unfortunately, there was a lack of benchmarks for a range of tests for female soccer players, especially across different ages. This should be a focus for future research, in particular across youth and senior players, with researchers striving for multi club/centre studies. As participation in female soccer continues to grow at youth and senior levels, it is important that practitioners working in female soccer can design appropriate strength and conditioning programs to physically prepare players for the increasing demands with increasing age [89,7] and competition [8]. Finally, previous researchers have highlighted the need for high quality research to help address the greater injury risk in female soccer players [17,9,10,19], however only two studies were identified to assess injury risk. Future research should consider force plate assessment, once appropriate benchmarks have been developed, to prospectively follow injury risk.

## Supporting information

S1 File A 27-item checklist defined within the Preferred Reporting Items for Systematic Reviews and Meta-Analyses extension for Scoping Reviews (PRISMA-ScR). (DOCX)

S2 File Search terms with controlled vocabulary and key words across multiple search input boxes in PubMed, Medline, and CINAHL. (DOCX)
