# Supplementary material for: A scoping review of force plates in female soccer: The utility, existing practice and identification of knowledge Gaps
Source: PLoS One. 2026 Jun 15;21(6):e0351121. doi: 10.1371/journal.pone.0351121 (PMC13268157; doi:10.1371/journal.pone.0351121)
Supplement: S2 File — (DOCX) [file pone.0351121.s002.docx]

**Google Scholar**

"girl*" OR “female*” OR “women*” AND “football*” OR “soccer*” AND "force plat*" AND "jump" OR "isometric" AND (“multi joint*” OR “single joint*” OR “plantar flex*” OR “knee*” OR “hamstring*” OR “knee flex*” OR “hip exten*” OR “posterior chain*” OR "drop*" OR "rebound*" OR "stiffness*" "power*" OR "strength*" OR "counter*movement*" OR “dominant*” OR “non dominant*”)

**PubMed. CINHAL, MedLine**

"girl*" OR “female*” OR “women*” AND “football*” OR “soccer*”

AND "force plat*"

AND "jump" OR "isometric"

AND (“multi joint*” OR “single joint*” OR “plantar flex*” OR “knee*” OR “hamstring*” OR “knee flex*” OR “hip exten*” OR “posterior chain*” OR "drop*" OR "rebound*" OR "stiffness*" "power*" OR "strength*" OR "counter*movement*" OR “dominant*” OR “non dominant*”)

Outcome of PubMed, Cinhal and MedLine searches:

((("girl*" OR "female*" OR "women*" AND "football*" OR "soccer*") AND ("force plat*")) AND ("jump" OR "isometric")) AND ("multi joint*" OR "single joint*" OR "plantar flex*" OR "knee*" OR "hamstring*" OR "knee flex*" OR "hip exten*" OR "posterior chain*" OR "drop*" OR "rebound*" OR "stiffness*" "power*" OR "strength*" OR "counter*movement*" OR "dominant*" OR "non dominant*"))
